# ﻿Five new species of *Bradina* Lederer (Lepidoptera, Crambidae) from China, with remarks on the morphology of the genus

**DOI:** 10.3897/zookeys.1158.99411

**Published:** 2023-04-19

**Authors:** Jia-Ming Guo, Xi-Cui Du

**Affiliations:** 1 College of Plant Protection, Southwest University, Chongqing, China Southwest University Chongqing China

**Keywords:** Genitalia, identification key, morphology, Pyraloidea, Spilomelinae, taxonomy

## Abstract

*Bradina* is a species-rich genus that differs from most other Spilomelinae genera because of its distinctive wing venation. Most species of this genus are very similar in appearance. In this study, we have studied morphological characteristics of the genus and eight closely related species from China. Among them, *B.falciculata* Guo & Du, **sp. nov.**, *B.fusoidea* Guo & Du, **sp. nov.**, *B.spirella* Guo & Du, **sp. nov.**, *B.ternifolia* Guo & Du, **sp. nov.** and *B.torsiva* Guo & Du, **sp. nov.** are described as new to science. *Bradinamegesalis* (Walker, 1859), *B.translinealis* Hampson, 1896 and *B.subpurpurescens* (Warren, 1896) are redescribed based on their holotypes and additional material, and the latter two are newly recorded from China and their genitalia are described for the first time. The images of the habitus and genitalia of these eight species are provided, with a key to their identification.

## ﻿Introduction

*Bradina* Lederer, 1863 is the most species-rich genus in the Spilomelinae tribe Steniini Guenée, 1854, redefined by Mally et al. (2019). So far, there are 89 species recorded in this genus worldwide (Nuss et al. 2003–2023) and they are mainly distributed in the Oriental and Australian regions. Numerous endemic species are present in the Australian and Pacific islands.

In Spilomelinae, *Bradina* can be differentiated from most other genera by the forewings with Rs_1_ anastomosed with Rs_2_+s_3_ at the base, which is common in Acentropinae. Therefore, this genus was placed in Hydrocampinae (= Acentropinae Stephens, 1835) for a long time (Hampson 1896; Hampson 1897; Rothschild 1915; Schaus 1924; Caradja 1925). Inoue (1955) transferred the genus to Pyraustinae (s. l.). Systematics research on *Bradina* is inadequate globally besides some early studies (Hampson 1896; Hampson 1897; Yamanaka 1984; Du 2008). Seizmair (2021) recorded *Bradina* from the Arabian Peninsula and divided the genus into seven groups according to wing pattern.

Species identification of *Bradina* is difficult because of their very similar appearance, so the genitalia characteristics are necessary in the identification of most species. The large spinose crescent-shaped signum of the female genitalia is a diagnostic characteristic of the genus, but shows little difference among species. In male genitalia, the valvae and uncus are very diverse in morphology, which is very valuable for interspecific identification. Before this study, 13 species were recorded in China (Lu and Guan 1953; Wang and Speidel 2000; Du 2008). In the present study, eight *Bradina* species with externally similar adults and male genitalia morphology are recorded, including five new species and two newly recorded species from China.

## ﻿Materials and methods

Specimens examined, including the types of new species, are deposited in the College of Plant Protection, Southwest University, Chongqing, China (**SWU**) except for two holotypes and 38 paratypes which are deposited in the Insect Collection of the College of Life Science, Nankai University, Tianjin, China (**NKU**). The corresponding author examined many specimens of *Bradina* deposited in Natural History Museum, London, United Kingdom (**NHMUK**), including some types.

The photographs of the adults were taken with a digital camera (Canon EOS 5D), and those of the genitalia were obtained with a digital camera (Leica DFC 450) attached to a stereomicroscope (Leica M205 A).

The preparation of genitalia mainly follows Li and Zheng (1996). Morphological terminology mainly refers to Maes (1995) as well as Mally and Nuss (2011).

## ﻿Taxonomic account

### ﻿ Bradina

Taxon classificationAnimaliaLepidopteraCrambidae

Lederer, 1863

28965585-2DCA-5E71-9B33-F3EFEEF2D287

Bradina Lederer, 1863: 424. Type species: Bradinaimpressalis Lederer, 1863; subsequent designation by Hampson 1896.
Erilita Lederer, 1863: 426. Type species: Erilitamodestalis Lederer, 1863, by monotypy.
Pleonectusa Lederer, 1863: 426. Type species: Botysadmixtalis Walker, 1859; subsequent designation by Moore 1884.
Trematarcha Meyrick, 1886: 233. Type species: Marasmiaerilitalis Felder, Felder & Rogenhofer, 1875; subsequent designation by Klima 1937.

#### Diagnosis.

**Figure 1.**
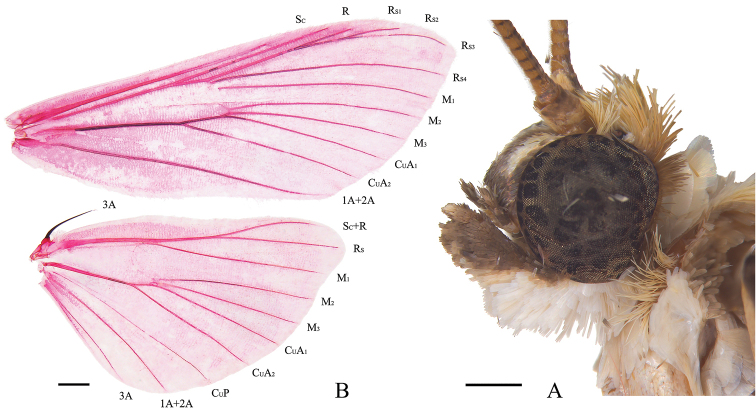
*Bradinamegesalis*, male **A** head **B** wing venation, wing slide no. GJM21001. Scale bars: 0.5 mm (**A**); 1.0 mm (**B**).

***Head*** (Fig. 1A). Frons rounded. Antenna annulated, male with short cilia ventrally. Labial palpus obliquely upturned, second segment with broad scales ventrally, third joint minute and forward, apex blunt. Maxillary palpus filiform. ***Thorax*.** Forewing long and narrow usually; length of cell c. half of wing; discocellulars incurved; R from cell at c. four fifths above; Rs_1_ anastomosed with Rs_2_+s_3_ at base and with a long stalk c. two fifths of Rs_3_; Rs_2_ and Rs_3_ stalked c. three fifths of Rs_3_; basal half of Rs_4_ straight and clearly separated from Rs_1_+s_2_+s_3_; M_2_, M_3_ and CuA_1_ uniformly from posterior angle of cell at base (except for *B.diagonalis* Hampson, 1896); CuA_2_ from cell at three quarters below. Hindwing with length of cell c. one third of wing; discocellulars incurved; Sc+R and Rs stalked c. one third of Rs; Rs and M_1_ from anterior angle of cell; M_2_, M_3_ and CuA_1_ uniformly from posterior angle of cell; CuA_2_ from cell at four fifths below (Fig. 1B). Legs long and slender; middle tibia with outer distal spur c. half-length of inner spur. ***Abdomen*.** Male abdomen long and slender (except for *B.melanoperas* Hampson, 1896).

***Male genitalia*.** Uncus diverse, apex with setae dorsally. Valva narrow or broad, some with well-developed setal cluster. Saccus developed. Phallus long and cylindrical.

***Female genitalia*.** Papillae anales densely setose. Apophyses anteriores c. twice length of apophyses posteriores. Ostium bursae well developed. Corpus bursae rounded or oval, inside usually densely studded with tiny spines; signum crescent and densely spinose, spines on concave side developed.

#### Remarks.

The generic characteristics were summarized by Khan (2000), but the description about wing venation was incomplete. In addition, the lengths of the maxillary palpus and labial palpus were identical in Khan’s description, while we found that both have their ends at the same height but they are of different lengths. Therefore, the generic characteristics are revised in the present study.

The bodies of *Bradina* species are usually brown, yellowish brown, or pale brown, except for a few species with white bodies, and have nearly identical wing markings. The male genitalia, on the other hand, are very diverse among species of this genus. We found that the male genitalia can be divided into three types according to the morphological characteristics of the valvae. The first type, represented by *B.admixtalis*, have long and narrow valvae; the second type, represented by *B.melanoperas*, have short and broad valvae; the third type, represented by *B.megesalis*, have broad valvae in which the costa is arched near the base or middle. Species in the present study have the third valva type, accompanied by the following common characteristics: body brown of various shades. Uncus broad, distal part bilobed, with dense short setae dorsally. Valva broad; costa arched near base or middle and accompanied by a cluster of long setae, usually followed by a depression. Saccus nearly trapezoidal, slightly concaved terminally. Juxta nearly rounded, split posteriorly. Phallus distinctly inflated at anterior end. Apophyses anteriores expanded at c. one third from base.

##### ﻿Key to *Bradina* species in the present study based on genitalia

**Table:** 

1	Sacculus with a cluster of long setae near middle	**2**
–	Sacculus without long setal cluster	**5**
2	Phallus with one fusiform cornutus composed of short and blunt spines, and one subcircular sclerotized cornutus	***B.fusoidea* sp. nov.**
–	Phallus with various cornuti, but not as above	**3**
3	Phallus with three long leaf-like cornuti tapered apically	***B.ternifolia* sp. nov.**
–	Cornuti not as above	**4**
4	Posterior phallus with two developed spear-like cornuti	***B.megesalis***
–	Phallus with one short and spiral band-like cornutus tapered at posterior end, along with two lamellar cornuti in posterior half	***B.spirella* sp. nov.**
5	Valva nearly rectangular, broad distally	***B.subpurpurescens***
–	Valva nearly elliptical or narrowed distally	**6**
6	Phallus with one broad and spiral band-like cornutus	***B.torsiva* sp. nov.**
–	Phallus with cornutus not as above	**7**
7	Phallus with one fusiform cornutus medially, and with two lamellar cornuti posteriorly. Ductus bursae slender, membranous	***B.translinealis***
–	Phallus with one small fusiform cornutus anteriorly, and with two sickle-shaped cornuti posteriorly. Ductus bursae relatively thick, slightly sclerotized medially	***B.falciculata* sp. nov.**

### ﻿ Bradina megesalis

Taxon classificationAnimaliaLepidopteraCrambidae

(Walker, 1859)

AD684363-FB4E-576B-8A13-550285D65AB4

Figs 1A , 2A , 3A–E

Botys megesalis Walker, 1859: 663. Type locality: North China. Type depository: NHMUK.
Bradina megesalis : Hampson, 1897: 200.

#### Material examined.

***Holotype***, ♂ **North China**, from Mr Fortune’s collection (Walker, 1859), genitalia slide no. 8735 (NHMUK).

#### Additional material.

**China, Chongqing Municipality**, 2 ♂♂, 6 ♀♀, Bashan Town, Chengkou County, alt. 900 m, 10 July 2017, Ji-Ping Wan leg.; 2 ♂♂, 4 ♀♀, Jinfo Mountain, alt. 918 m, 27 August 2019, You Zeng leg.; 1 ♂, Simian Mountain, alt. 1280 m, 12 July 2012, Gui-Qing He leg.; **Guangdong Prov.**, 2 ♂♂, 2 ♀♀, Dadong Mountain, Lian County, 5–8 July 2008, Feng-Xia He leg., genitalia slide no.: GJM21078 ♂; 1 ♂, 2 ♀♀, Qianjin Conservation Station, Shimentai Nature Reserve, Qingyuan City, alt. 523 m, 26 May 2021, Xing-Hai Zuo leg., genitalia slide no.: GJM21073 ♂; **Guangxi Zhuang Autonomous Region**, 1 ♂, Yinshan Park, Dayao Mountain, alt. 1564 m, 8 July 2013, Xiao-Hua Chen leg.; **Guizhou Prov.**, 2 ♂♂, 4 ♀♀, Leigong Mountain, Leishan County, alt. 1198 m, 14–15 July 2013, Xiao-Hua Chen leg., genitalia slide no.: GJM21076 ♂; **Hubei Prov.**, 18 ♂♂, 33 ♀♀, Dabie Mountain, alt. 590 m, 24–25 June 2014, Li-Jun Xu leg.; **Hainan Prov.**, 1 ♂, 1 ♀, Bawangling National Forest Park, 8–10 June 2010, Li Kang leg.; **Hunan Prov.**, 5 ♀♀, Wuyunjie Nature Reserve, alt. 178 m, 19 June 2019, Ying Yang leg.; **Sichuan Prov.**, 2 ♂♂, 1 ♀, Longcanggou Forest Park, Xingjing County, alt. 1388 m, 17 June 2021, Shuai Yu leg., genitalia slide no.: GJM21074 ♂, GJM21080 ♀; **Shaanxi Prov.**, 1 ♂, Zuoshui County, Shangluo City, alt. 810 m, 29 June 2021, Jin-Hang Han leg.; 1 ♂, 5 ♀♀, Hanyin County, Ankang City, alt. 410 m, 26 June 2021, Jin-Hang Han leg.; **Yunnan Prov.**, 9 ♂♂, 6 ♀♀, Baihualing Village, Baoshan City, alt. 1487 m, 20–23 June 2020, Ying Yang & Hong Zhao leg.; 2 ♂♂, 1 ♀, Cuanlong Village, Mangba Town, Tengchong City, Baoshan City, alt. 1329 m, 8 August 2015, Jing-Xia Zhao & Hao Wei leg.; **Zhejiang Prov.**, 6 ♂♂, 7 ♀♀, Jiulongshan Forest Park, 4–6 August 2011, Xiao-Bing Fu leg.; 6 ♂♂, 34 ♀♀, Tianmu Mountain, alt. 800 m, 29 July 2011, Xi-Cui Du & Xiao-Bing Fu leg.

#### Redescription.

**Figure 2.**
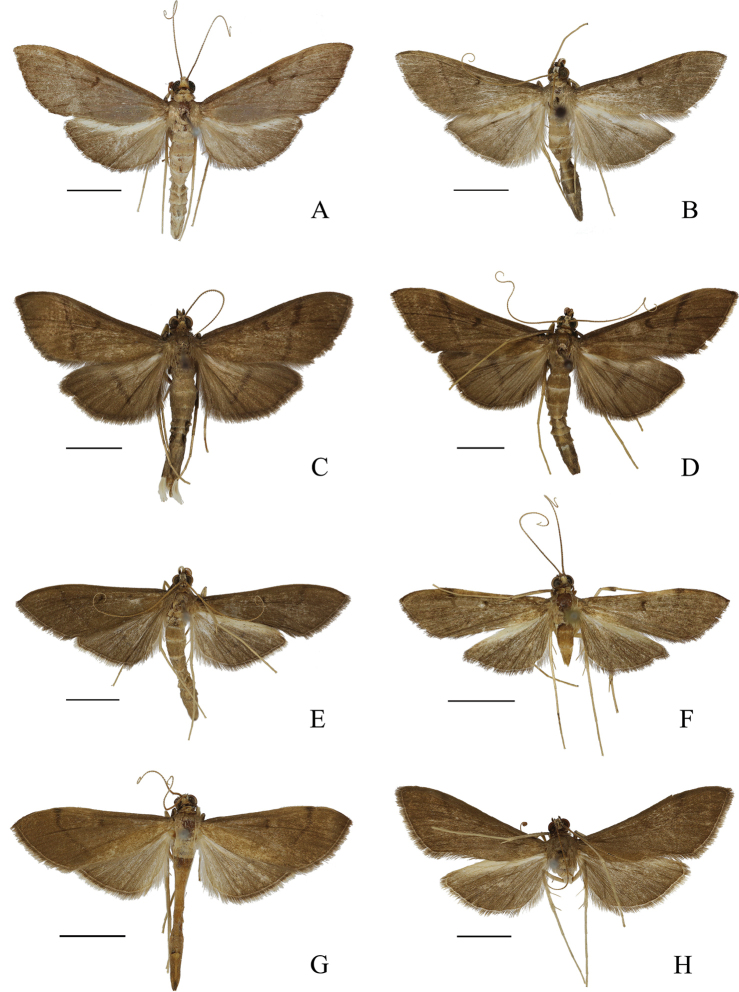
Habitus of *Bradina* species, male **A***B.megesalis***B***B.fusoidea* sp. nov., holotype **C***B.spirella* sp. nov., holotype **D***B.torsiva* sp. nov., holotype **E***B.subpurpurescens***F***B.falciculata* sp. nov., holotype **G***B.translinealis***H***B.ternifolia* sp. nov., holotype. Scale bars: 0.5 cm.

***Adult*** (Figs 1A, 2A). Wingspan 31.0–39.0 mm, forewing length 15.0–19.0 mm. Body and wings pale brown. Frons brown, with lateral sides white above. Vertex yellowish white. Antenna brownish yellow, with black ring dorsally; ventral cilia c. one quarter length of diameter of male flagellomeres. Labial palpus with basal two thirds white, black-brown distally. Maxillary palpus yellowish brown, white at base. Patagium pale brown, yellowish. Thorax white ventrally. Forewing pale brown, darker along basal two thirds costa, stigmata and lines brown; discoidal stigma crescent; postmedial line at c. two thirds of wing, straight and nearly parallel to terminal margin. Hindwing with postmedial line and discoidal stigma pale brown, inconspicuous usually; discoidal stigma crescent. Cilia pale brown, yellowish white on inner margin of hindwing. Legs pale yellow, coxae and femora with white gloss. Middle tibia brown; hind tibia with outer middle spurs c. two thirds length of inner spurs. Abdomen pale brown dorsally, with each segment pale terminally; yellowish white ventrally.

**Figure 3.**
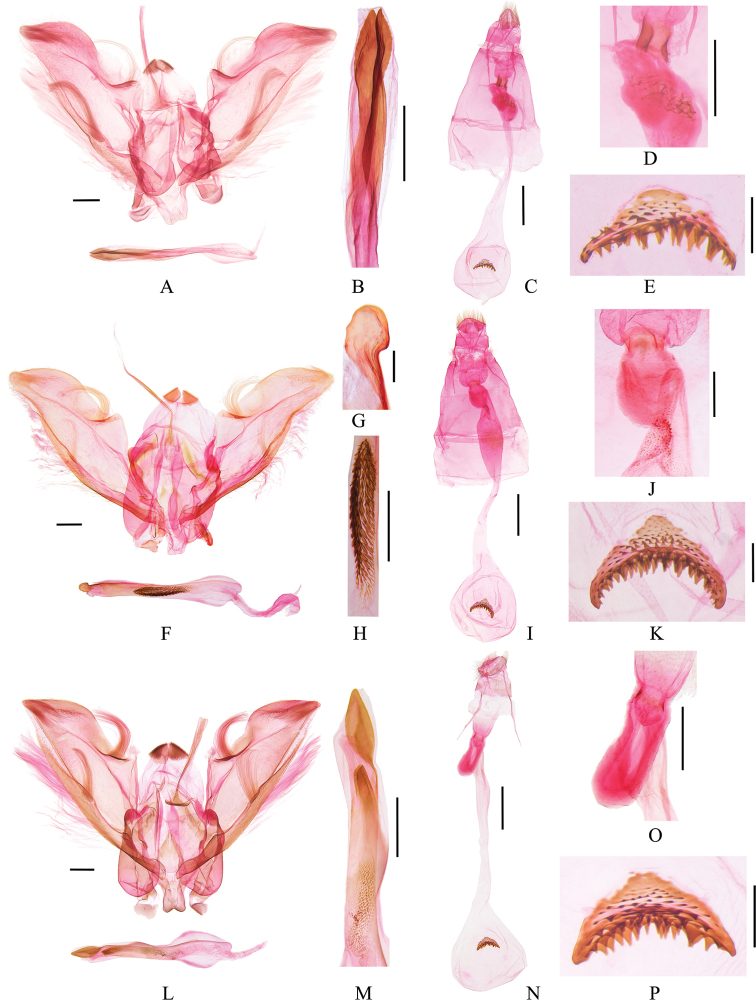
Genitalia of *Bradina* species **A–E***B.megesalis***A, B** male, slide no. GJM21074 **C–E** female, slide no. GJM21080 **F–K***B.fusoidea* sp. nov. **F–H** male holotype, slide no. GJM21117 **I–K** female, paratype, slide no. GJM21118 **L–P***B.spirella* sp. nov. **L, M** male, holotype, slide no. GJM21161 **N–P** female, paratype, slide no. GJM21164 **B, G, H, M** partial enlargement of phallus **D, J, O** partial enlargement of ductus bursae **E, K, P** signum. Scale bars: 0.2 mm (**E, G, K, P**); 0.5 mm (**A, B, D, F, H, J, L, M, O**); 1.0 mm (**C, I, N**).

***Male genitalia*** (Fig. 3A, B). Valva distally gradually narrowed and bearing dense long setae; costa sharply arched near middle and accompanied by a cluster of long curved setae; sacculus gradually narrowed to apex, with a cluster of long setae near middle. Posterior phallus with two developed spear-like cornuti.

***Female genitalia*** (Fig. 3C–E). Antrum broad. Ductus bursae posteriorly sharply inflated into a thorny irregular protrusion, adjoined posteriorly by crescent colliculum laterally, anterior half gradually widened to corpus bursae. Corpus bursae nearly rounded, with dense tiny spines inside, transverse signum crescent and densely spinose.

#### Distribution.

China (Chongqing, Fujian, Gansu, Guangdong, Guangxi, Guizhou, Hubei, Hainan, Hunan, Sichuan, Shaanxi, Yunnan, Zhejiang), Japan (Shibuya 1929).

#### Remarks.

We found that the coremata of some male individuals of this species were protruded out of the body, forming a cluster of white hairs at the end of the abdomen.

### ﻿ Bradina fusoidea

Taxon classificationAnimaliaLepidopteraCrambidae

Guo & Du sp. nov.

EFD38EBC-E110-5E97-95BF-2921FDB9F170

https://zoobank.org/CEACA386-3CD0-4FFF-85F5-1140F4B86FEF

Figs 2B , 3F–K

#### Type material.

***Holotype***, ♂ **China: Sichuan Prov.**, Qingcheng Mountain, Dujiangyan city, alt. 860 m, 30°92'N, 103°50'E, 22 July 2021, Shuai Yu, Xiao-Ju Zhu, Di Zhang leg. (NKU), genitalia slide no. GJM21117. ***Paratypes*. China: Sichuan Prov.**, 6 ♂♂, 1 ♀, other same data as holotype (NKU), genitalia slide no.: GJM21112 ♂, GJM21118 ♀; 1 ♂, Emei Mountain, Leshan City, alt. 847 m, 22 July 2021, Shuai Yu, Xiao-Ju Zhu, Di Zhang leg. (NKU), genitalia slide no.: GJM21113; **Guangdong Prov.**, 2 ♂♂, 1 ♀, Heishiding Nature Reserve, Fengkai County, Zhaoqing City, 14–16 June 2009, Feng-Xia He leg., genitalia slide no.: GJM21114 ♂, GJM21180 ♀.

#### Diagnosis.

This species is similar to *B.megesalis*. The difference in appearance is that the distance between discoidal stigma and postmedial line on forewing of this species is longer than that of *B.megesalis*; the postmedial line and terminal margin of forewing is obviously unparallel in this species, while is nearly parallel in the latter. It also can be distinguished by the setal cluster near middle of sacculus being shorter than that of the latter, phallus with one fusiform cornutus and one subcircular cornutus (two spear-like cornuti in *B.megesalis*), posterior ductus bursae inflated but not forming irregular protrusion as in *B.megesalis*, plus subposterior section of ductus bursae widened along half of ductus length, a feature that is absent in *B.megesalis*.

#### Description.

***Adult*** (Fig. 2B). Wingspan 31.0–33.0 mm, forewing length 15.0–16.0 mm. Frons white, except brown on frontal base and middle near vertex. Vertex white. Antenna yellow, with pale brown ring dorsally; ventral cilia c. half-length of flagellomere diameter of male. Labial palpus with basal two thirds white, black-brown distally. Maxillary palpus black-brown. Patagium yellowish white. Tegula pale brown. Thorax pale brown dorsally, white ventrally. Wings pale brown, gradually darkened to terminal; stigmata and lines dark brown. Forewing black-brown along basal half of costa; orbicular stigma very small; discoidal stigma crescent; postmedial line at c. two thirds of wing, straight and unparallel to terminal margin. Hindwing with postmedial line straight, towards to tornus, only middle part obvious. Cilia pale brown, with a white line at base, except yellowish white on inner margin of the hindwing. Legs pale yellow. Front and middle tibiae brown; hind tibia with outer middle spurs c. two thirds length of inner spurs. Abdomen with basal half pale brown and distal half dark brown dorsally, each segment pale terminally; yellowish white ventrally.

***Male genitalia*** (Fig. 3F–H). Valva distally gradually narrowed and bearing dense long setae; costa arched near middle and accompanied by a cluster of long curved setae; sacculus gradually narrowed to apex, with a cluster of long setae near middle. Phallus with one fusiform cornutus medially, composed of short and blunt spines, c. one third length of phallus, and one subcircular sclerotized cornutus posteriorly.

***Female genitalia*** (Fig. 3I–K). Antrum broad. Posterior ductus bursae inflated and thorny, adjoined posteriorly by colliculum, posterior half inflated, weakly sclerotized, narrowed medially, then gradually widened to corpus bursae. Corpus bursae nearly rounded, with dense tiny spines inside, transverse signum crescent and densely spinose.

#### Etymology.

The specific name is derived from the Latin *fusoideus* (meaning ‘fusiform’), in reference to a fusiform cornutus.

#### Distribution.

China (Guangdong, Sichuan).

### ﻿ Bradina spirella

Taxon classificationAnimaliaLepidopteraCrambidae

Guo & Du sp. nov.

D793159B-2637-5522-998B-7B17A7320853

https://zoobank.org/2AF3F855-1103-42AA-94DD-D6634879BB67

Figs 2C , 3L–P

#### Type material.

***Holotype***, ♂ **China: Hunan Prov.**, Xianchijie, Wuyunjie National Nature Reserve, alt. 720 m, 28°90'N, 111°48'E, 24 June 2019, Ying Yang leg., genitalia slide no. GJM21161. ***Paratypes*. China: Hunan Prov.**, 9 ♂♂, 4 ♀♀, Bamian Mountain Nature Reserve, Guidong County, alt. 973 m, 16 June 2015, Kai Chen leg., genitalia slide no.: GJM21160 ♂; 5 ♂♂, 2 ♀♀, Zhushan Village, Wuyunjie National Nature Reserve, alt. 100 m, 15 June 2019, Ying Yang leg.; 34 ♂♂, 14 ♀♀, Jindongjie, Wuyunjie National Nature Reserve, alt. 178 m, 16–19 June 2019, Ying Yang leg.; 66 ♂♂, 22 ♂♂, 18–24 June 2019, other same data as holotype, genitalia slide no.: GJM21048 ♂, GJM21049 ♂, GJM21162 ♂, GJM21163 ♂, GJM21164 ♀; **Jiangxi Prov.**, 4 ♂♂, 2 ♀♀, Jinggang Mountain, 30 June 2011, Jin-Wei Li leg., genitalia slide no.: GJM21159 ♂, GJM21183 ♀.

#### Diagnosis.

This species is similar to *B.megesalis*. The difference in appearance is that wings of this species are darker in color and hindwings are slightly broader; the postmedial line and terminal margin of forewing are obviously unparallel in this species, while nearly parallel in the latter. It also can be distinguished by phallus with one short, spiral band-like cornutus and two lamellar cornuti, and some tiny spines on the vesica medially; the posterior ductus bursae is inflated into a thick finger-like protrusion. In *B.megesalis*, the phallus has two spear-like cornuti and is without spines on the vesica; the posterior ductus bursae is inflated into an irregular protrusion.

#### Description.

***Adult*** (Fig. 2C). Wingspan 29.0–32.0 mm, forewing length 14.0–15.5 mm. Frons brown, with lateral sides yellowish white above. Vertex pale yellow. Antenna brownish yellow, with black-brown ring dorsally, basal segments of flagellum black-brown dorsally; ventral cilia c. one third length of flagellomere diameter of male. Labial palpus with basal two thirds white, black-brown distally. Maxillary palpus black-brown. Patagium and tegula dark brown. Thorax dark brown dorsally, white ventrally. Wings dark brown, stigmata and lines black-brown. Forewing black-brown along costa, slightly paler distally; orbicular stigma very small; discoidal stigma crescent; postmedial line at c. two thirds of wing, inconspicuously waved and unparallel to terminal margin. Hindwing pale at base; postmedial line straight, only middle part obvious. Cilia greyish brown. Legs pale yellow. Front and middle tibiae dark brown; hind tibia with outer middle spurs c. same length as inner spurs. Abdomen brown dorsally, with each segment pale terminally; yellowish white ventrally.

***Male genitalia*** (Fig. 3L, M). Valva with the distal part gradually narrowed and bearing dense long setae; costa arched near middle and accompanied by a cluster of long curved setae; sacculus gradually narrowed to apex, with a cluster of long setae near middle. Juxta narrowed distally. Phallus with one short and spiral band-like cornutus tapered at posterior end, along with two lamellar cornuti in posterior half; some tiny spines on vesica medially.

***Female genitalia*** (Fig. 3N–P). Antrum broad. Ductus bursae slender, posteriorly inflated into a thick finger-like protrusion, adjoined posteriorly by colliculum. Corpus bursae nearly oval, without spines inside, transverse signum crescent and densely spinose.

#### Etymology.

The specific name is derived from the Latin *spirellus* (meaning ‘small spiral-like’), in reference to a short and spiral band-like cornutus.

#### Distribution.

China (Hunan, Jiangxi).

#### Remark.

Coremata of some male individuals of this species were protruded out of the body, forming a cluster of white hairs at the end of the abdomen.

### ﻿ Bradina torsiva

Taxon classificationAnimaliaLepidopteraCrambidae

Guo & Du sp. nov.

C0DF853D-5753-5882-804A-6DE644BAE48E

https://zoobank.org/3DF230C3-6BA8-4D7F-AB54-61C46C1CE689

Figs 2D , 4A–E

#### Type material.

***Holotype***, ♂ **China: Hunan Prov.**, Chenzhou Nature Reserve, alt. 1233 m, 25°78'N, 113°01'E, 3 June 2019, Xiao-Qiang Lu & Ying Yang leg., genitalia slide no. GJM21102. ***Paratypes*. China: Hunan Prov.**, 4 ♂♂, other same data as holotype, genitalia slide no.: GJM21103 ♂; 1♂, Zhushan Village, Taoyuan County, Changde City, alt. 100 m, 15 June 2019, Xiao-Qiang Lu & Ying Yang leg., genitalia slide no.: GJM21101; 2 ♂♂, Maozhu River, Shimen County, Changde City, alt. 350 m, 6 June 2017, Jian-Yue Qiu & Hao Xu leg.; **Guangdong Prov.**, 2 ♂♂, 1 ♀, Dadong Mountain, Lianzhou City, alt. 650 m, 21 June 2004, Dan-Dan Zhang leg., genitalia slide no.: GJM21100 ♂; 1 ♀, Qingyuan City, alt. 270 m, 7 June 2019, Xiao-Qiang Lu & Ying Yang leg.; 1 ♂, 2 ♀♀, Babaoshan Conservation Station, Nanling National Nature Reserve, alt. 980 m, 19 May 2021, Xing-Hai Zuo leg. (NKU), genitalia slide no.: GJM21099 ♂; 5 ♂♂, 2 ♀♀, Yangmeikeng Village, Shimentai National Nature Reserve, alt. 870 m, 27 May 2021, Xing-Hai Zuo leg. (NKU), genitalia slide no.: GJM21104 ♀.

#### Diagnosis.

This species is similar to *B.megesalis*. The difference in appearance is that wings of this species are darker in color, and hindwings are slightly broader; the postmedial line and terminal margin of forewing is obviously unparallel in this species, while it is nearly parallel in the latter. It also can be distinguished by the sacculus without long setal cluster, the phallus with one broad, spiral, band-like cornutus; the posterior third of ductus bursae with slightly sclerotized elongate inflation, but not forming irregular protrusion. In *B.megesalis*, the sacculus has long setal cluster near middle, and the phallus has two spear-like cornuti; the posterior ductus bursae is inflated into an irregular protrusion.

#### Description.

***Adult*** (Fig. 2D). Wingspan 36.0–37.0 mm, forewing length 17.5–18.0 mm. Frons brown, with lateral sides yellowish white above. Vertex yellowish white. Antenna brownish yellow, with black-brown ring dorsally, ventral cilia c. half-length of flagellomere diameter of male. Labial palpus with basal two thirds white, black-brown distally. Maxillary palpus black-brown or brown. Patagium yellowish white. Tegula dark brown. Thorax dark brown dorsally, white ventrally. Wings dark brown, stigmata and lines black-brown. Forewing black-brown along costa, slightly paler distally; orbicular stigma very small; discoidal stigma crescent; postmedial line at c. two thirds of wing, unparallel to terminal margin. Hindwing slightly pale at base; postmedial line inconspicuously waved, only middle part obvious. Cilia pale yellow, with a black-brown line at base, except black-brown on inner margin. Legs yellow. Front and middle tibiae dark brown; hind tibia with outer middle spurs c. three fifths length of inner spurs. Abdomen brown dorsally, each segment pale terminally; paler ventrally.

**Figures 4.**
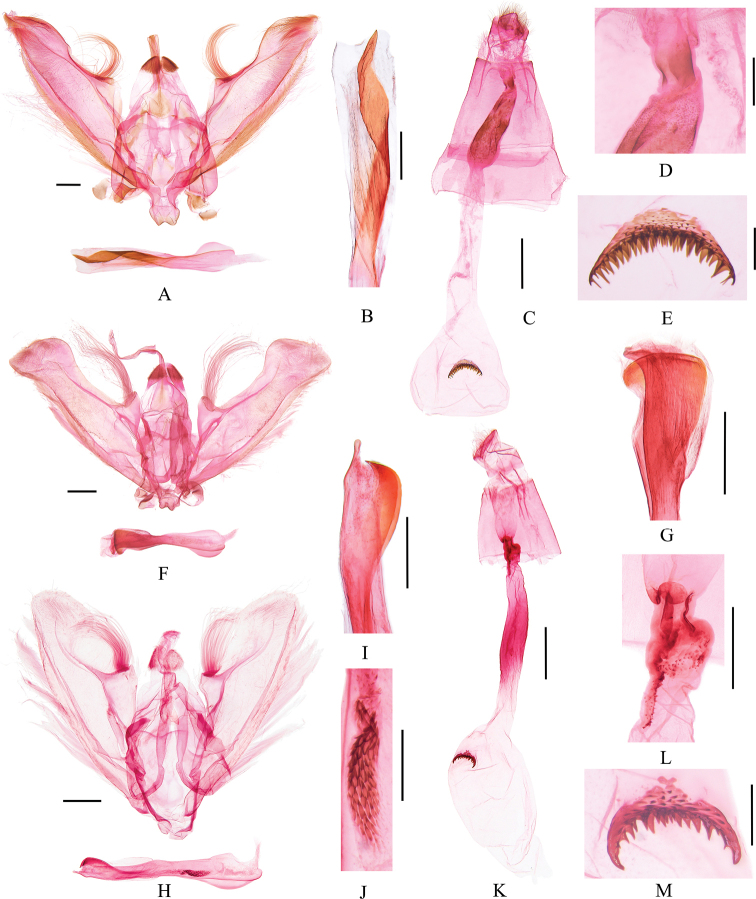
Genitalia of *Bradina* species **A–E***B.torsiva* sp. nov. **A, B** male, holotype, slide no. GJM21102 **C–E** female, paratype, slide no. GJM21104 **F, G***B.subpurpurescens* male, slide no. GJM21018 **H–M***B.falciculata* sp. nov. **H–J** male, holotype, slide no. GJM21084 **K–M** female, paratype, slide no. GJM21085 **B, G, I, J** partial enlargement of phallus **D, L** partial enlargement of ductus bursae **E, M** signum. Scale bars: 0.2 mm (**D, E, J, M**); 0.5 mm (**A, B, F–I, L**); 1.0 mm (**C, K**).

***Male genitalia*** (Fig. 4A, B). Valva with the distal part gradually narrowing and with dense long setae; costa arched near middle and accompanied by a cluster of long curved setae; sacculus narrowed distally, without long setal cluster. Juxta narrowed distally. Phallus slightly inflated, with one broad and spiral band-like cornutus posteriorly.

***Female genitalia*** (Fig. 4C–E). Ductus bursae broad, with posterior third inflated, sclerotized, and thorny, adjoined posteriorly by colliculum, gradually widened to corpus bursae. Corpus bursae nearly oval, truncated terminally, with dense tiny spines inside, transverse signum crescent and densely spinose.

#### Etymology.

The specific name is derived from the Latin *torsivus* (meaning 'spiral'), in reference to a spiral band-like cornutus.

#### Distribution.

China (Guangdong, Hunan).

#### Remark.

Coremata of some male individuals of this species were protruded out of the body, forming a cluster of white hairs at the end of the abdomen.

### ﻿ Bradina subpurpurescens

Taxon classificationAnimaliaLepidopteraCrambidae

(Warren, 1896)

4C59DAC2-1922-5371-A656-4F854F5CBEA6

Figs 2E , 4F, G

Pleonectusa subpurpurescens Warren, 1896: 147. Type locality: India. Type depository: NHMUK.
Bradina subpurpurescens : Hampson, 1896: 227.

#### Material examined.

***Holotype***, ♂ **India**: Khasis, X. 1894, Nat. Coll (NHMUK).

#### Additional material.

**China: Yunnan Prov.**, 1 ♂, Meizihu Park, Simao District, Pu’er City, alt. 1400 m, 11 May 2018, Xi-Cui Du & Xiao-Qiang Lu leg., genitalia slide no.: GJM21019; 1 ♂, Yunpan Mountain, Pu’er City, alt. 1400 m, 9 July 2013, Zhen-Guo Zhang leg., genitalia slide no.: GJM21018; 1 ♂, Taiyanghe National Forest Park, Puer City, alt. 1659 m, 29 June 2021, Yao Shen & Ci Tang leg., genitalia slide no.: GJM21020.

#### Redescription.

***Adult*** (Fig. 2E). Wingspan 26.0–33.0 mm, forewing length 14.5–16.0 mm. Frons brown, with lateral sides white above. Vertex brownish yellow. Antenna brownish yellow, with pale brown ring dorsally; length of ventral cilia c. one quarter of the flagellomere diameter of male. Labial palpus with basal two thirds white, black-brown distally. Maxillary palpus black-brown, pale brown at base. Patagium and tegula brown. Thorax pale brown distally, white ventrally. Wings dark brown, stigmata and lines black-brown. Forewing black-brown along basal costa; orbicular stigma very small; discoidal stigma crescent; postmedial line at c. three quarters of wing, nearly parallel to terminal margin, slightly excurved near costa. Hindwing with postmedial line slightly beyond basal half of wing, only middle part obvious. Cilia pale brown on forewing, yellowish white on hindwing; a brown line at base. Legs pale yellow. Front and middle tibiae yellowish brown; hind tibia with outer middle spurs c. half-length of inner spurs. Abdomen pale yellowish brown dorsally, each segment pale terminally.

***Male genitalia*** (Fig. 4F, G). Valva nearly rectangular, broad distally; costa sharply arched near base and accompanied by a cluster of long curved setae, then slightly concave towards apex; sacculus gradually narrowed distally, without long setal cluster. Phallus narrow medially, significantly inflated anteriorly and posteriorly, posterior half sclerotized; cornutus absent.

#### Distribution.

China (Yunnan), India.

#### Remarks.

This species is recorded in China for the first time, and its male genitalia are also described for the first time. It can be distinguished from the other species (except *B.falciculata*) in the present study by the forewing relatively narrower; cilia pale brown on forewing, yellowish white on hindwing. The female of this species is unknown.

### ﻿ Bradina falciculata

Taxon classificationAnimaliaLepidopteraCrambidae

Guo & Du sp. nov.

783F4B0E-C6CB-5ABD-8860-EAE2ACC2B086

https://zoobank.org/9F1E4111-1E29-42C2-AD1D-300EAC54E761

Figs 2F , 4H–M

#### Type material.

***Holotype***, ♂ **China: Tibet Autonomous Region**, Medog County, alt. 1100 m, 29°32'N, 95°33'E, 14 August 2003, Xin-Pu Wang & Huai-Jun Xue leg. (NKU), genitalia slide no. GJM21084. ***Paratypes*. China: Tibet Autonomous Region**, 5 ♂♂, 3 ♀♀, other same data as holotype, genitalia slide no.: GJM21082 ♂; 5 ♂♂, 8 ♀♀, Bomi-Medog Highway, Medog County, alt. 880 m, 14 August 2003, Xin-Pu Wang & Huai-Jun Xue leg. (NKU), genitalia slide no.: GJM21083 ♂, GJM21085 ♀, GJM21086 ♀.

#### Diagnosis.

This species is similar to *B.subpurpurescens*. The difference in appearance is that wings are paler in color and the postmedial line of the forewing is placed at two thirds from the wing base in this new species, but at three quarters in the latter. It also can be distinguished by an elliptical valva and the phallus inconspicuously inflated distally. In *B.subpurpurescens*, the valva is nearly rectangular and the phallus is significantly inflated distally.

#### Description.

***Adult*** (Fig. 2F). Wingspan 25.0–27.0 mm, forewing length 12.0–13.0 mm. Frons brown, with lateral sides yellowish white above. Vertex yellowish white. Antenna yellow, with pale brown ring dorsally; ventral cilia c. half-length of flagellomere diameter of male. Labial palpus with basal two thirds white, dark brown distally. Maxillary palpus dark brown. Patagium and tegula pale brown. Thorax pale brown dorsally, white ventrally. Wings yellowish brown, stigmata and lines brown. Forewing with discoidal stigma crescent; postmedial line at basal two thirds of wing, slightly excurved and nearly parallel to terminal margin. Hindwing with postmedial line straight, usually not obvious. Cilia pale brown, with a darker line at base, except greyish white on inner margin of the hindwing. Legs pale yellow. Front and middle tibiae black-brown on distal half; hind tibia with outer middle spurs c. half-length of inner spurs. Abdomen pale brown dorsally, yellowish white ventrally.

***Male genitalia*** (Fig. 4H–J). Valva elliptical; costa arched near middle and accompanied by a cluster of long curved setae; sacculus gradually narrowed to apex, without long setal cluster. Juxta narrowed distally. Phallus with one small fusiform cornutus anteriorly, composed of short and blunt spines, c. one sixth length of phallus, and with two sickle-shaped cornuti posteriorly.

***Female genitalia*** (Fig. 4K–M). Antrum broad. Ductus bursae with one oval sclerotized piece close to crescent-shaped colliculum, then inflated and thorny, slightly sclerotized medially. Corpus bursae nearly oval, with dense tiny spines inside, transverse signum crescent and densely spinose.

#### Etymology.

The specific name is derived from the Latin *falciculatus* (meaning 'falcate'), in reference to two sickle cornuti of posterior phallus.

#### Distribution.

China (Tibet).

### ﻿ Bradina translinealis

Taxon classificationAnimaliaLepidopteraCrambidae

Hampson, 1896

1CFF027D-6FB2-587A-A17A-23891E86FB3C

Figs 2G , 5A–F

Bradina translinealis Hampson, 1896: 228. Type locality: N. W. Himalayas. Type depository: NHMUK.

#### Material examined.

***Holotype***, ♂. Moore Coll. 94–106, genitalia slide no. 8734. (NHMUK).

#### Additional material.

**China, Yunnan Prov.**, 5 ♂♂, 2 ♀♀, Baihualing Village, Baoshan City, alt. 1520 m, 11–13 August 2007, Dan-Dan Zhang leg., genitalia slide no.: GJM21165 ♂, GJM21166 ♂, GJM21167 ♀, GJM21182 ♀.

#### Redescription.

***Adult*** (Fig. 2G). Wingspan 25.0–30.0 mm, forewing length 12.0–13.0 mm. Frons brown, with lateral sides yellowish white above. Vertex yellowish white. Antenna brownish yellow, with brown ring dorsally; ventral cilia c. quarter length of flagellomere diameter of male. Labial palpus with basal two thirds white, black-brown distally. Maxillary palpus black-brown, yellowish white at base. Patagium and tegula pale brown. Thorax pale brown dorsally, white ventrally. Forewing yellowish brown, dark brown along basal half of costa; stigmata and lines black-brown; discoidal stigma reniform; postmedial line at c. two thirds of wing, slightly excurved near costa. Hindwing pale yellowish brown dorsally, yellowish white to pale brown ventrally; paler at base; postmedial line pale brown, inconspicuous, extending beyond basal half of wing. Cilia pale brown on forewing, yellowish white on hindwing, a brown line at base. Legs yellowish white. Front and middle tibiae brown; hind tibia with outer middle spurs c. half-length of inner spurs. Abdomen pale yellowish brown dorsally, yellowish white ventrally.

**Figure 5.**
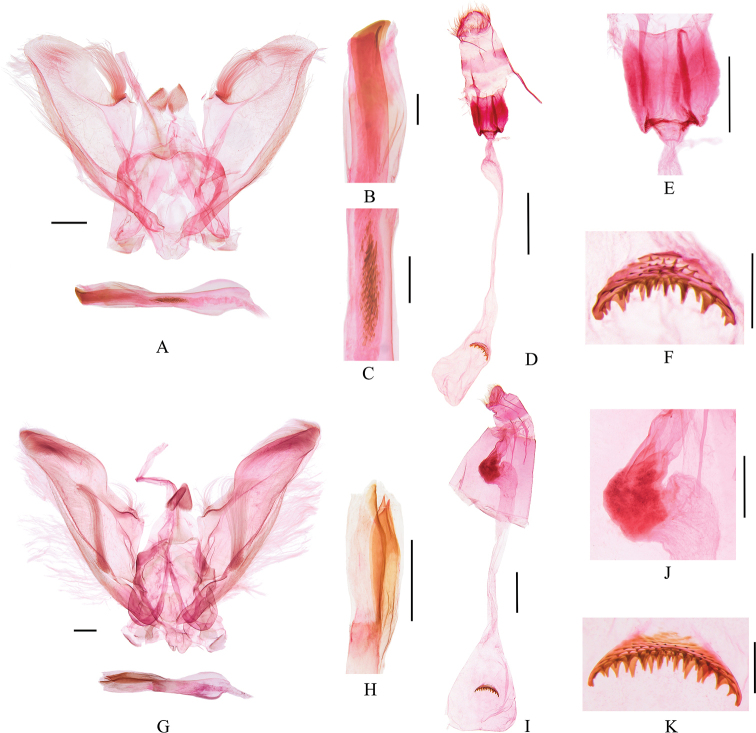
Genitalia of *Bradina* species **A–F***B.translinealis***A–C** male, slide no. GJM21165 **D–F** female, slide no. GJM21167 **G–K***B.ternifolia* sp. nov. **G, H** male, holotype, slide no. GJM21173 **I–K** female, paratype, slide no. GJM21178 **B, C, H** partial enlargement of phallus **E, J** partial enlargement of ductus bursae **F, K** signum. Scale bars: 0.2 mm (**B, C, F, K**); 0.5 mm (**A, E, G, H, J**); 1.0 mm (**D, I**).

***Male genitalia*** (Fig. 5A–C). Valva nearly elliptical, bearing dense long setae distally; costa arched near middle and accompanied by a cluster of long setae; sacculus gradually narrowed distally, without long setal cluster. Phallus with one fusiform cornutus medially, composed of short and blunt spines, c. one fifth length of phallus; posterior half slightly inflated, and with two weakly sclerotized lamellar cornuti posteriorly.

***Female genitalia*** (Fig. 5D–F). Antrum broad and sclerotized, strongly sclerotized laterally, anteriorly adjoined by colliculum. Posterior ductus bursae slender, inflated, widened close to corpus bursae. Corpus bursae nearly oval, truncated terminally; with dense tiny spines inside, transverse signum crescent and densely spinose.

#### Distribution.

China (Yunnan), N. W. Himalayas.

#### Remarks.

This species is first recorded in China and its genitalia are described for the first time. It can be distinguished by the valva being nearly elliptical, the phallus with one fusiform cornutus medially and two weakly sclerotized lamellar cornuti posteriorly.

### ﻿ Bradina ternifolia

Taxon classificationAnimaliaLepidopteraCrambidae

Guo & Du sp. nov.

DBFAEF32-B750-58DA-BC55-3487AC8E4E3F

https://zoobank.org/F49AC3BF-5605-4538-A1AF-145EA7B517FF

Figs 2H , 5G–K

#### Type material.

***Holotype***, ♂ **China: Yunnan Prov.**, Dahaoping Village, Tengchong City, 25°02'N, 98°49'E, 6 August 2007, Dan-Dan Zhang leg., genitalia slide no. GJM21173. ***Paratypes*. China: Yunnan Prov.**, 2 ♂♂, 7 ♀♀, other same data as holotype, genitalia slide no.: GJM21174 ♂, GJM21175 ♂, GJM21176 ♀, GJM21177 ♀, GJM21178 ♀, GJM21181 ♀; 2 ♀♀, 5 August 2007, other same data as holotype.

#### Diagnosis.

This species is similar to *B.translinealis*. It can be distinguished by postmedial line of forewing straight; valva elongated and narrowed comparatively, sacculus with long setal cluster near middle, posterior phallus with three long leaf-like cornuti; antrum membranous. In *B.translinealis*, postmedial line of forewing slightly excurved near costa; sacculus has no setal cluster, phallus has one fusiform cornutus medially and two lamellar cornuti posteriorly; antrum broader and sclerotized.

#### Description.

***Adult*** (Fig. 2H). Wingspan 31.0–33.0 mm, forewing length 15.0–16.0 mm. Frons brown, except black-brown above. Vertex yellowish white mixed with brownish yellow. Antenna pale yellow, with pale brown ring dorsally; ventral cilia c. half-length of flagellomeres diameter of male. Labial palpus with basal half yellowish white, brown or black-brown distally. Maxillary palpus brown or black-brown. Patagium and tegula dark brown. Thorax dark brown dorsally, white ventrally. Wings brown, stigmata and lines black-brown. Forewing black-brown along costa; discoidal stigma reniform; postmedial line at c. two thirds of wing, straight and nearly parallel to terminal margin. Hindwing with discoidal stigma crescent; postmedial line beyond basal half of wing, usually inconspicuous. Cilia pale brown on forewing, greyish white on hindwing, a darker line at base. Legs pale yellow. Front and middle tibiae brown; hind tibia with outer middle spurs c. three fifths length of inner spurs. Abdomen dark brown dorsally, each segment pale terminally; slightly pale ventrally.

***Male genitalia*** (Fig. 5G, H). Valva gradually narrowed and bearing dense long setae distally; costa arched near base and accompanied by a cluster of short and curved setae; sacculus gradually narrowed to apex, with a cluster of long setae near middle. Posterior phallus inflated slightly, with three long leaf-like cornuti tapered apically.

***Female genitalia*** (Fig. 5I–K). Antrum membranous. Ductus bursae slender, adjoined posteriorly by crescent colliculum laterally, then inflated and bent. Corpus bursae nearly oval, with dense tiny spines inside, transverse signum crescent and densely spinose.

#### Etymology.

The specific name is derived from the Latin *ternifolius* (meaning 'trifoliate'), in reference to three long leaf-like cornuti.

#### Distribution.

China (Yunnan).

#### Remark.

Coremata of some male individuals of this species were protruded out of the body, forming a cluster of white hairs at the end of the abdomen.

## ﻿Discussion

The species in the present study, represented by *B.megesalis*, have a broad valva, whose costa is arched near the base or middle and accompanied by a cluster of long curved setae. The cornuti of the phallus of these species are diverse and therefore useful for interspecific identification. Seizmair (2021) divided *Bradina* into seven groups according to wing pattern characteristics, such as forewing and hindwing of the same color or of different colors, and whether the postmedial line is absent or present, and in the latter case whether it is straight or curved. In our opinion, however, it is difficult to divide the genus based only on wing pattern and color, and it would be more convincing to divide it by combining the appearance and morphology of the genitalia. In addition, most species of the *B.diagonalis* group in Seizmair’s study are similar to the species in the present study both in appearance and genitalia. However, we found that *B.diagonalis*, as representative species of the *B.diagonalis* group, is different from most other species of this group in wing venation and genitalia. Therefore, further study on more species globally and in more detail is needed to clarify this confusion. In addition, the transverse crescent signum of *Bradina* is a distinctive feature. Like *Bradina*, the Steniini genera *Diathrausta* and *Perisyntrocha* also have an arched, transverse signum in the female genitalia’s corpus bursae (Mally et al. 2019). This could indicate an evolutionary relatedness of these three genera, which is expected to be addressed in the future research.

## Supplementary Material

XML Treatment for Bradina

XML Treatment for Bradina megesalis

XML Treatment for Bradina fusoidea

XML Treatment for Bradina spirella

XML Treatment for Bradina torsiva

XML Treatment for Bradina subpurpurescens

XML Treatment for Bradina falciculata

XML Treatment for Bradina translinealis

XML Treatment for Bradina ternifolia
